# Coordinative interaction of microcrystalline chitosan with oxovanadium (IV) ions in aqueous solution

**DOI:** 10.1186/s13065-014-0050-7

**Published:** 2014-09-09

**Authors:** Marta E Lichawska, Kazimiera H Bodek, Julia Jezierska, Aleksander Kufelnicki

**Affiliations:** Department of Physical and Biocoordination Chemistry, Faculty of Pharmacy, Medical University of Łódź, 90-151 Łódź, Poland; Chair of Applied Pharmacy, Faculty of Pharmacy, Medical University of Łódź, 90-151 Łódź, Poland; Faculty of Chemistry, University of Wrocław, 50-383 Wrocław, Poland

**Keywords:** Biomaterial, Microcrystalline chitosan, Vanadium (IV), Metal-polymer complexes, Equilibria in aqueous solution

## Abstract

**Background:**

Chitosan, a non-toxic, biodegradable and biocompatible polysaccharide has attained great interest in pharmaceutical applications, as versatile drug delivery agent. Chitosan has been already shown to serve as vehicle for sustained drug release by chitosan-vanadium(IV) complex from a chitosan gel matrix. Therefore, chitosan gel proved to retain vanadium and preserve its insulin-mimetic efficacy. Nevertheless, there is a lack of reports concerning complexing equilibria in aqueous solution, in particular when using the more advantageous microcrystalline form of chitosan (MCCh). Microcrystalline chitosan shows a number of valuable features as compared with unmodified chitosan.

**Results:**

Experimental studies on complexing interaction between a special form of biomaterial - microcrystalline chitosan as ligand, L = MCCh, of two exemplary degrees of deacetylation DD (lower 79.8%; higher 97.7%) with M = oxovanadium (IV) ions have been carried out potentiometrically at four ligand-to-metal concentration ratios (2:1, 5:1, 8:1, 10:1). Among the five hydrolysis equilibria of VO^2+^ reported up to now in the literature, under the conditions of the present work i.e. aqueous solutions of ionic strength *I* = 0.1 (KNO_3_) and temperature 25.0 ± 0.1°C, the predominating one was (VO)_2_(OH)_2_^2+^ formation: log *β*_20–2_ = −7.01(2). Analysis of potentiometric results permitted to note that degree of deacetylation does not essentially influence the coordination mode of the complexes formed. In the case of both the two DD values, as well as for all the ligand-to-metal ratios, formation of hydroxyl deprotonated MLH_−1_ and ML_2_H_−2_ moieties has been confirmed potentiometrically (log *β*_11–1_ = −0.68(2) for DD = 79.8% and −0.68(2) for DD = 97.7%, log *β*_12–2_ = −7.64(6) for DD = 79.8% and −5.38(7) for DD = 97.7%).

**Conclusion:**

Microcrystalline chitosan coordinates the vanadyl ions by the hydroxyl groups. Interaction of MCCh with VO^2+^ ions in aqueous solution occurs within pH 5–7. Amounts of alkali excessive towards -NH_2_ are needed to deprotonate the OH groups. Deprotonation occurring at the chitosan hydroxyl groups permits a “pendant” or “bridge” model of coordination with VO(IV). Lack of complexation via deprotonation of amine groups, typical for simple cations and the molybdenum anion, has been indicated also by FTIR spectroscopy and EPR.

**Graphical Abstract:**
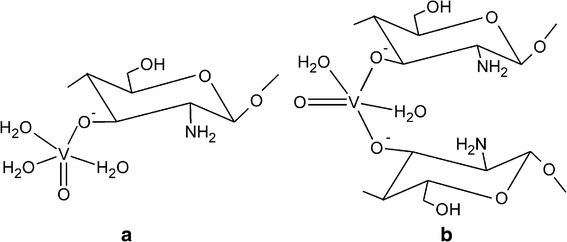
Coordination modes of VO(IV) with microcrystalline chitosan (MCCh): (a)- pendant model, (b)- bridge model

## Background

**Figure 1:**
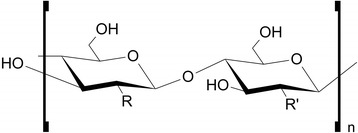
**Structure of neutral chitosan (R = −NH**_**2**_ **, R’ = −NHOC-CH**_**3**_ **.** In chitosan R predominates, in chitin every substituent is R’).

Chitosan is a polysaccharide composed of D–glucosamine (2–amino–2–deoxy–β-D–glucose) and *N*-acetyl-D-glucosamine (2–acetamido–2–deoxy–β-D–glucose) (Figure 1). This biopolymer is formed by partial deacetylation of chitin, the most widespread natural polysaccharide, found in shells of crustaceans. The molar D-glucosamine to *N*-acetyl-D-glucosamine ratio in the chitosan sample is an estimate of the so called degree of deacetylation (DD). The term chitosan is referred to chitin of DD > 60% [1-3]. The degree of deacetylation of the copolymer is usually within the range of 70.0% - 95.0% [4-5].

This non-toxic, biodegradable and biocompatible material has attained great interest in pharmaceutical applications, as versatile drug delivery agent [1-3]. A valuable physicochemical modification of chitosan, used among others as excipient in drug formulations, is microcrystalline chitosan (MCCh), obtained in form of suspension, powder or granules [6-7]. A polymer of desired chemical properties is prepared by appropriate aggregation of the macromolecules from aqueous solutions of organic acids (via neutralization, coagulation and then precipitation of microcrystalline chitosan) [8]. During this process the polymer is also refined from low-molecular side products. Microcrystalline chitosan shows a number of valuable features as compared with unmodified chitosan: higher absorptivity, chelating capability, higher bioactivity, as well as ability of forming polymer films directly from water slurry. Similarly as it was the case with the previous forms of chitosan, MCCh may be proposed as an effective vehicle for the controlled vanadium release [3].

Vanadium (IV) plays a number of roles in the physiology of living systems. It is responsible for a number of processes, e.g. the inhibition of phosphate–metabolising enzymes such as phosphatases, ribonuclease and ATP-ases. The interaction of VO^2+^ with phosphates and nucleotides is of special interest in vanadium biochemistry [9]. The insufficiency of vanadium may, among other effects, lead to lowering the level of erythrocytes and pathologic increase of lipid level in blood. Recently, the medical significance of this element focuses on enhancing the treatment of type I diabetes mellitus (insulin-dependent) and also of type II (noninsulin-dependent) [10]. However, direct application of vanadium by vanadyl sulphate has several undesired side-effects, such as gastrointestinal symptoms, anorexia and weight loss. Hence, it is necessary to elaborate a delivery mechanism suitable to be used in therapy. The most successful clinical trials were carried out with bis(maltolato)oxonanadium(IV) compounds (BMOV - bis-3-hydroxy-4-pyronato)oxovanadium(IV) – and BEOV - bis(2-ethyl)-3-hydroxy-4-pyronato)oxovanadium(IV) but also with corresponding complexes of kojic acid (Hkoj - 3-hydroxy-6-hydroxymethyl-4-pyrone) and tropolone (Htrop - 2-hydroxy-2,4,6-cycloheptatrien-1-one) [11-15]. The first of them, BMOV, proved to be three times more effective than vanadyl sulfate as a glucose lowering agent. Recently also a number of other synthetic imidazole and pyridine based compounds of potential insulin-mimetic activity have been studied in solution [16-17]. Vanadium(IV) compounds mimic most of the biological effects of insulin in various cell types. They have been shown to increase glucose transport and oxidation (by that lowering glucose level in blood), to stimulate glycogen synthesis and to inhibit hepatic gluconeogenesis [18]. The anti-diabetic effect is probably attained via irreversible strong inhibition of the insulin receptor – one of the large class of tyrosine kinase receptors.

Unmodified chitosan has been already shown to serve as vehicle for sustained drug release by controlling the diffusion of chitosan-vanadium complex from a chitosan gel matrix. Therefore, chitosan gel proved to retain vanadium and preserve its insulin-mimetic efficacy [10]. Some earlier, kinetic and equilibrium studies on adsorption of VO^2+^ by chitosan were performed as well [19]. Up to now, the spectroscopic (only FTIR) investigation of the VO^2+^ - unmodified chitosan interaction was carried out for the final solid complex in comparison with pure chitosan [2]. Nevertheless, there is a lack of reports concerning complexing equilibria in aqueous solution, in particular when using the more advantageous microcrystalline form of chitosan (MCCh). Corresponding reactions of MCCh have been studied with other metal-ions in our previous papers [20-22]. It would be also interesting to investigate the DD influence on VO^2+^ - MCCh complex formation. This effect has already been discussed in our latest paper with Mo(VI) [23].

## Results and discussion

### Analysis of potentiometric data and speciation models

The pH potentiometric data were elaborated in order to obtain appropriate speciation models and to determine the stability constants of complexes.

### Hydrolysis of VO^2+^

The potentiometric studies of the vanadyl solution under exactly the same conditions as they were used in the potentiometric measurements with MCCh, described in later section, confirmed predomination of only the (VO)_2_(OH)_2_^2+^ hydroxo complex from among the species mentioned in the literature:

1 $$ 2{\mathrm{VO}}^{2+}+2{\mathrm{H}}_2\mathrm{O}\rightleftharpoons {\left(\mathrm{VO}\right)}_2{{\left(\mathrm{OH}\right)}_2}^{2+}+2{\mathrm{H}}^{+} \log {\beta}_{20-2}=-7.01(2) $$

The hydrolysis constant obtained was in good agreement with the corresponding corrected values reported recently: −6.95 [16, 17], and in fairly agreement with the elder data: −6.67 [24], -6.72 [25], −6.88 [26]. On the other hand the simplest VO(OH)^+^ hydroxo complex has been shown to occur in minor share up to pH ~ 5, just like it was indicated in ref. [19]. The remaining hydroxo species reported in the literature: [VO(OH)_3_]^−^ and [(VO)_2_(OH)_5_]^−^ occur at pH > 8 [16], that is to say beyond the pH range of complexation with MCCh.

### Complex formation with VO^2+^

**Figure 2:**
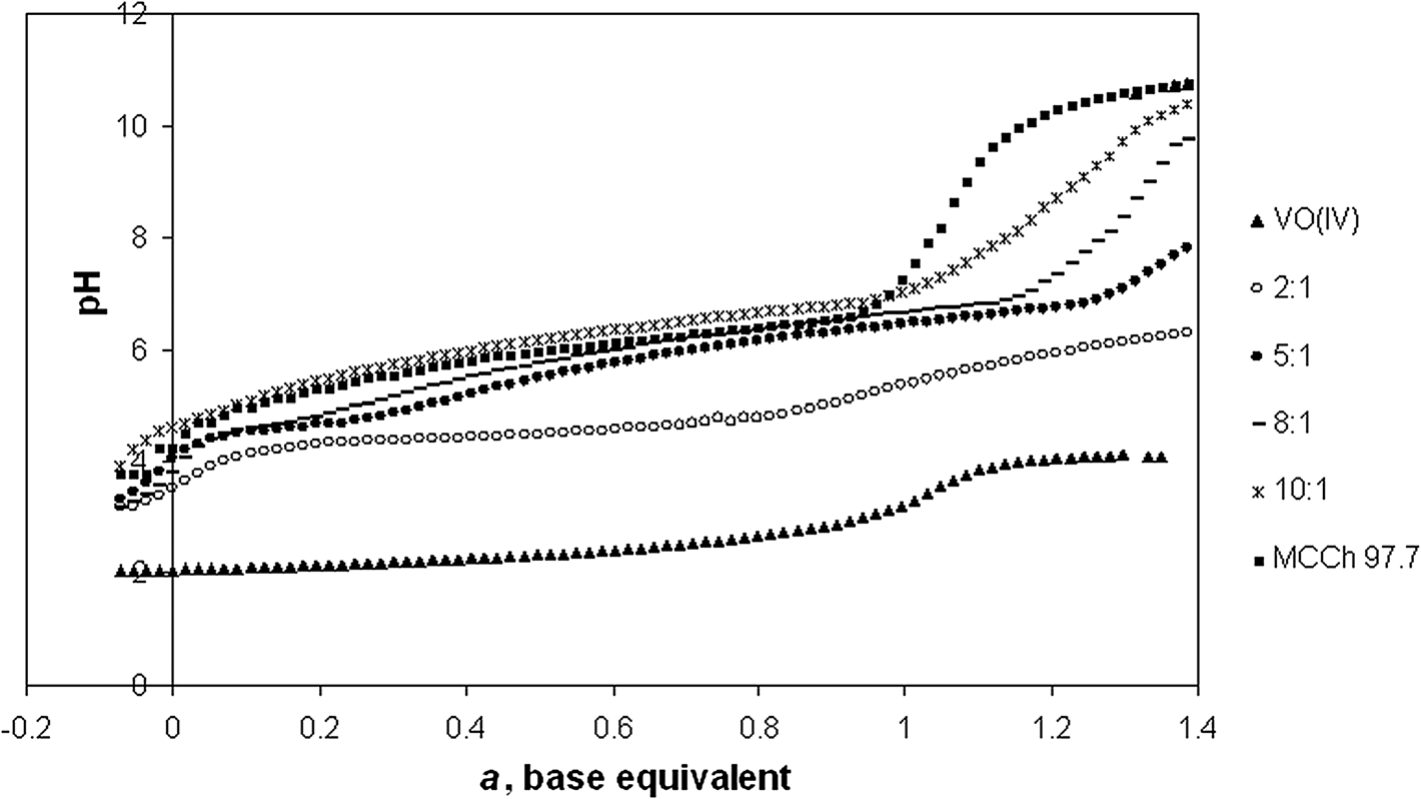
**Titration of the H** ^**+**^ **- VO(IV) – MCCh (DD = 0.977) system at various ligand to metal ratio.** C_MCCh_ (mol L^−1^) = 7.0 × 10^−3^. The value of base equivalent *a* = −0.1 corresponds to HNO_3_ in excess as related to ligand.

The potentiometric titrations in presence and absence of L = MCCh corresponded to neutralization of the hydrogen ions in dependence of base equivalent *a* (*a* = mmole of base/mmole of ligand). Measurements started with neutralization of the excessive mineral acid (*a* = −0.1 – 0) and then were continued by further alkalization of the –NH_3_^+^ chitosan groups up to *a* > 1 (Figure 2). As it follows also from Figure 2, the use of alkali needed to reach the end point in the samples with chitosan increased in presence of VO^2+^ - starting from ligand to metal ratio 10:1 up to 2:1. The values of pH were lower than for MCCh in absence of vanadyl ions. The parts of curves after *a* ~ 1 (end point of MCCh), the longer the higher is the part of vanadyl ion, are evidently connected with VO(IV) – MCCh complexation via the deprotonated hydroxyl groups but not the amine groups. This result is not surprising because it is known that the affinity of V^IV^O^2+^ ion is much higher towards ligands with oxygen donors [27-29]. Coordinative interaction of oxido V^IV^O^2+^ ion towards only nitrogen donors is very low and results in hydrolytic processes in acid and neutral solution. In turn, the titration curve corresponding to VO^2+^ in absence of ligand, showed a disturbance not far after the end point (*a* > ca 1.3), due to poor solubility of the forming aquo-hydroxo products.

Owing to the potentiometric results the proposed coordination reactions in the M = VO^2+^ – L = MCCh system are shown in Equations (2) and (3) – charges omitted for clarity.

2 $$ \begin{array}{l}\mathrm{M}+\mathrm{L}={\mathrm{MLH}}_{-1}+\mathrm{H}\hfill \\ {}{\beta}_{11-1}=\frac{\left[{\mathrm{MLH}}_{-1}\right]\left[\mathrm{H}\right]}{\left[\mathrm{M}\right]\left[\mathrm{L}\right]}\hfill \end{array} $$

3 $$ \begin{array}{l}\mathrm{M} + 2\ \mathrm{L} = {\mathrm{ML}}_2{\mathrm{H}}_{-2} + 2\ \mathrm{H}\hfill \\ {}{\beta}_{12-2}=\frac{\left[{\mathrm{ML}}_2{\mathrm{H}}_{-2}\right]{\left[\mathrm{H}\right]}^2}{\left[\mathrm{M}\right]{\left[\mathrm{L}\right]}^2}\hfill \end{array} $$

where: *β*_*mlh*_ = [M_*m*_L_*l*_H_*h*_]/[M]^*m*^[L]^*l*^[H]^*h*^ – cumulative stability constant; *m*, *l*, *h* – number of metals (central ions), ligands and protons, respectively; concentrations in square bracket are equilibrium concentrations.

Except of the hydrolysis reaction mentioned in Equation 1, the calculations involved the protonation constant of MCCh under the same conditions as in the present experiments with VO^2+^ (log *β*_011_ = 6.50, that is in accordance with our previous data [23] and also with the literature [30]).

**Figure 3:**
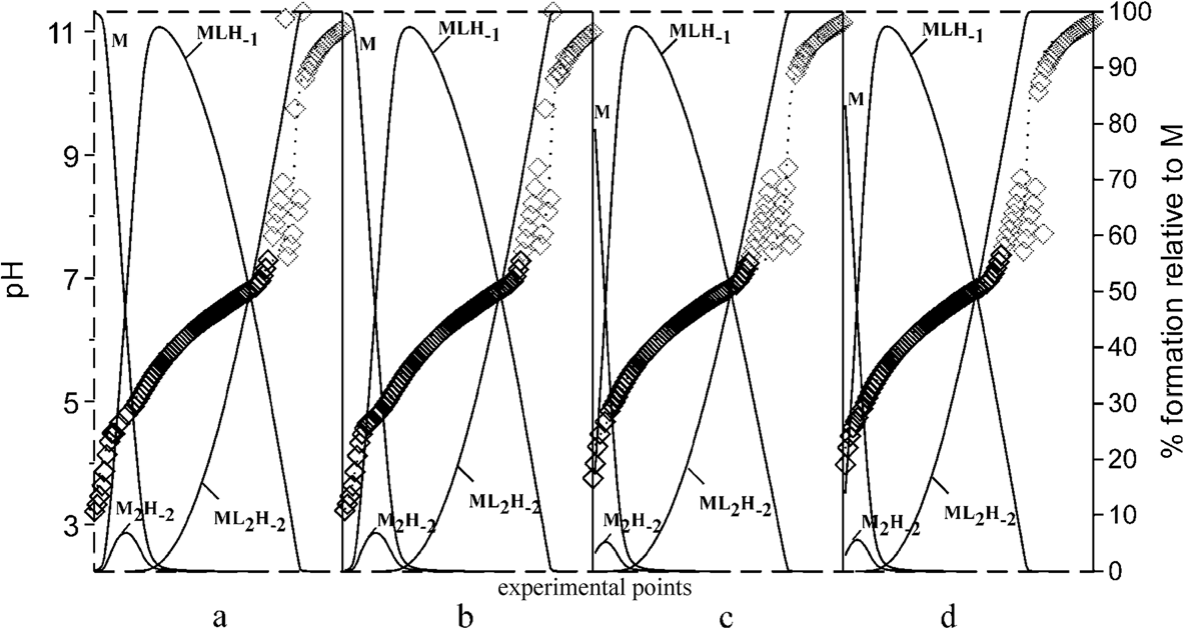
**Titration curves (presented by square points) and species distribution of the complexes formed in H** ^**+**^ **- VO(IV) – MCCh (DD = 0.977) system.** C_MCCh_ (mol L^−1^) = 7.0 × 10^−3^. Ligand to metal ratio **a)** 2:1, **b)** 5:1, **c)** 8:1, **d)** 10:1. Dotted points correspond to the pH range of visible precipitation.

**Figure 4:**
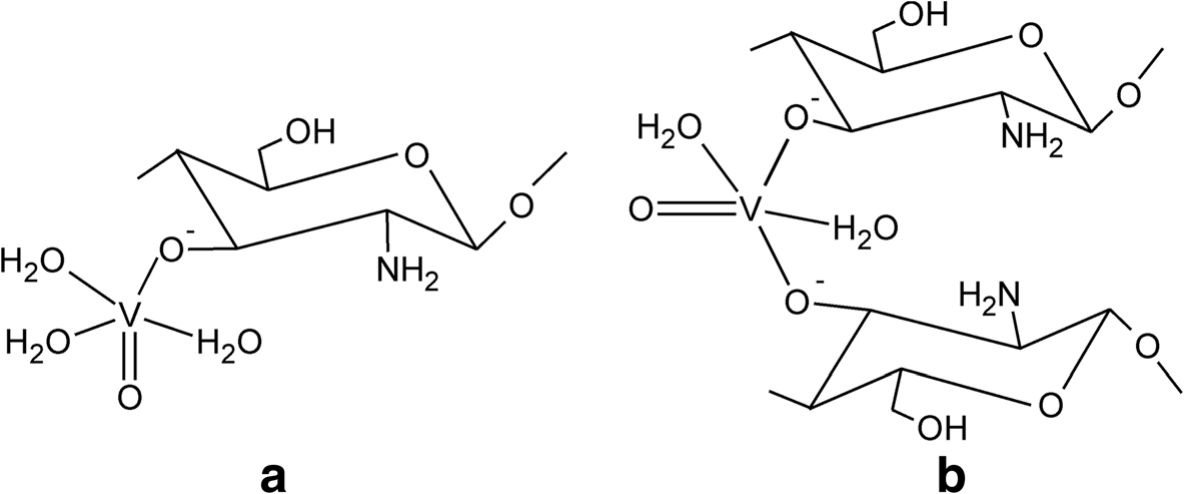
**Simplified coordination scheme of L = MCCh to M = oxovanadium(IV) at neutral pH: a) MLH**_**−1**_ **, b) ML**_**2**_ **H**_**−2**_ **.**

The next plot (Figure 3), directly originating from Hyperquad refinements, shows that the significant share of the accepted deprotonated species MLH_−1_ as well as lower share of the twice deprotonated ML_2_H_−2_ complex occurs at pH 5–7. At lower pH the refinements indicate a small contribution (up to 10%) of the M_2_H_−2_ = (VO)_2_(OH)_2_^2+^ hydroxo complex (Figure 3). In turn, at pH higher than ca 7 – 7.5, the increasing aggregation of chitosan makes impossible further accurate pH measurements. Finally, the present refinement results indicate donation via the deprotonated hydroxyl groups (Figure 4) just following –NH_3_^+^ deprotonation. On the other hand, the specific structure of chitosan as polymer chain with a long distance between consecutive mers suggests that the higher accessibility of coordination sites occurs for hydroxyl donors from two neighbouring polymer chains in the ML_2_H_−2_ complex (Figure 4b). The above mentioned pattern involving deprotonated OH groups bonded to the VO^2+^ cation has already been described for VO^2+^/carbohydrate complexes [9].

It is worthy to note that for the MLH_−1_ species the coordination mode does not essentially depend on the DD of chitosan (Table 1). However, at DD 97.7% the stability constant of the ML_2_H_−2_ species is somewhat higher (less negative) than for DD 79.8%. The latter difference is difficult to be interpreted because at pH > 7, i.e. at the end of the pH range used in calculations (and where the ML_2_H_−2_ species becomes predominating) as the titrations are evidently disturbed by increasing aggregation of MCCh of both DD’s.

**Table 1:** **Cumulative stability constants log** ***β***_***mlh***_^**a**^ **of the VO(IV) - MCCh complexes**

**Value of DD**	***C***_**L**_ **/** ***C***_**M**_	**log** ***β***_**11-1**_^**b**^	**log** ***β***_**11-1**_^**c**^	**log** ***β***_**12-2**_^**b**^	**log** ***β***_**12-2**_^**c**^	**pH range**
79.8%	2:1	−0.39 (5)	−0.68 (2)	−4.48 (23)	−7.64 (6)	2.53-7.34
		−0.26 (5)				
				−4.18 (24)		
	5:1	−0.82 (3)		−7.81 (16)		
		−0.80 (4)				
				−7.59 (8)		
	8:1	−0.90 (5)		−8.11 (10)		
		−0.87 (4)				
				−7.43 (10)		
	10:1	−0.99 (6)		−7.83 (26)		
		−0.98 (6)				
				−8.06 (11)		
97.7%	2:1	−0.53 (4)	−0.68 (2)	−4.67 (21)	−5.38 (7)	3.18-7.38
				−5.72 (29)		
		−0.56 (3)				
	5:1	−0.71 (4)		−4.41 (9)		
		−0.68 (4)		−4.45 (9)		
	8:1	−0.68 (4)		−4.95 (10)		
				−4.45 (9)		
		−0.88 (6)				
	10:1	−1.40 (3)		−6.76 (6)		
		−1.29 (8)				
				−5.69 (14)		

^a^refer to the general reaction: *m*(VO^2 +^) + *l*L + *h*H ⇌ (VO)_*m*_L_*l*_H_*h*_^2*m* + *h*^.

^b^results from individual titrations.

^c^results from comprehensive files of all titrations.

*T* = 25.0°C, *I* = 0.1 mol L^−1^ (KNO_3_). Standard deviations in parentheses. *C*_L_ = total concentration of ligand; *C*_M_ = total concentration of VO^2+^.

### Infrared spectra

**Figure 5:**
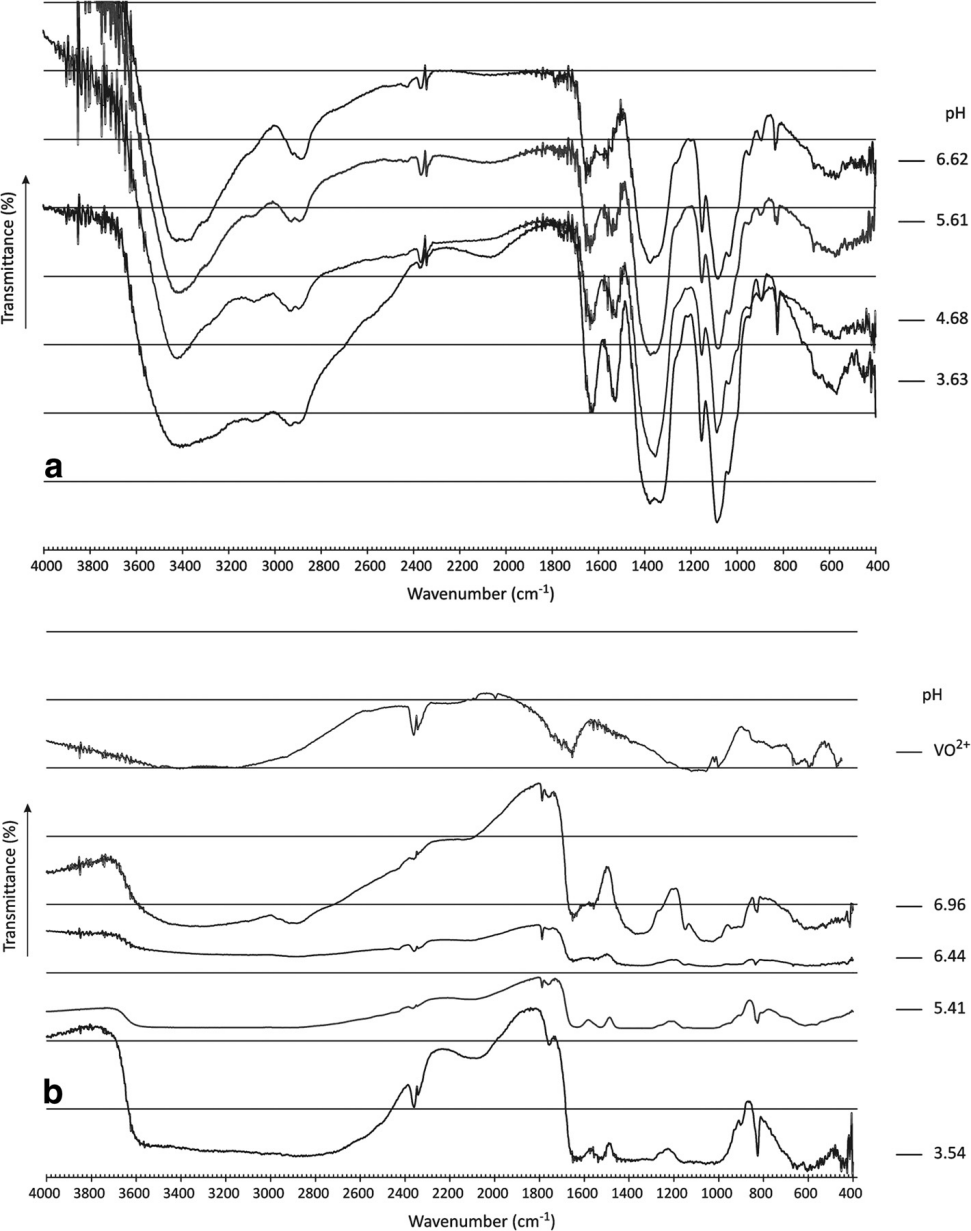
**FTIR spectra obtained in thin polymer films of a) pure MCCh (DD 79.8%) and b) MCCh in presence of VO** ^**2+**^ **ions at various pH.** Comparative spectrum for VO^2+^ taken in KBr pellets.

The FTIR spectra of free MCCh and of related MCCh samples in presence of VO^2+^ and at various pH are shown exemplary in Figures 5a and b for the lower DD value – 79.8%.

In free chitosan the broad band (due to hydrogen bond interaction) visible from the high wavenumber side of spectrum (~3400 cm^−1^) is assignable to the O-H and N-H stretching vibrations [2]. Moreover, the C-H stretching bands could be identified as doublets at 2900/2850 cm^−1^ (Figure 5a). In presence of VO(IV) the spectra become poorly unresolved within this range, most probably as a result of overlapping by the vanadyl ion line and also due to the lower thickness and higher fragility of the films obtained in presence of VO^2+^.

In the medium IR range 600 – 1800 cm^−1^ the region 1400–1800 cm^−1^ is of particular interest. Our spectra of pure MCCh derived from thin films, much more distinct that the ones recorded from KBr pellets, showed both the so called amide I band at ca 1630 cm^−1^ (which is essentially the C = O stretching vibration of the –NHOC-CH_3_ group) [2] as well as the amide II band of the secondary amide groups and the deformation scissoring mode, δ(NH_2_), of the primary amine groups –NH_2_ – ca 1530 cm^−1^. As can be observed the two bands are well splitted and free of noise up to pH at least 5.5. In presence of VO(IV) the rise of pH resulted in lowering and deformation of the latter bands, also probable due to progressive deprotonation of the –NH_3_^+^ and following aggregation of the MCCh chains – (ref. Figure 4b). The bands are only remaining in lowering intensity only at pH 3.54 – 6.44. Characteristic that in presence of VO(IV) the spectra do not show any visible shift of the amide II band nor of the scissoring mode of the primary amine groups, δ(NH_2_). Such shifts towards higher wavenumbers have been already described for interaction of chitosan with other metals, like Cu(II), Hg(II) and Ni(II) [31-32]. Hence, this observation may be evidently connected with our previously discussed potentiometric titrations confirming rather complexation via the hydroxyl oxygens than the amine nitrogens.

An additional spectral IR line of pure VO^2+^ was shown in Figure 5b. Its special feature may be assigned to the low intensive, scarcely splitted band at ca 1000 cm^−1^, due to the characteristic V = O stretching vibration [3-9]. The visible disappearance of this band in presence of MCCh (starting from the lowest pH 3.54, where potentiometry shows already the share of complexation) may be explained by strong hydrogen-bond interactions between free hydroxyl groups of the carbohydrate ligand and the oxo group of the metal center thus leading to diminution of the V = O bond strength.

### Indications of EPR spectroscopy

**Figure 6:**
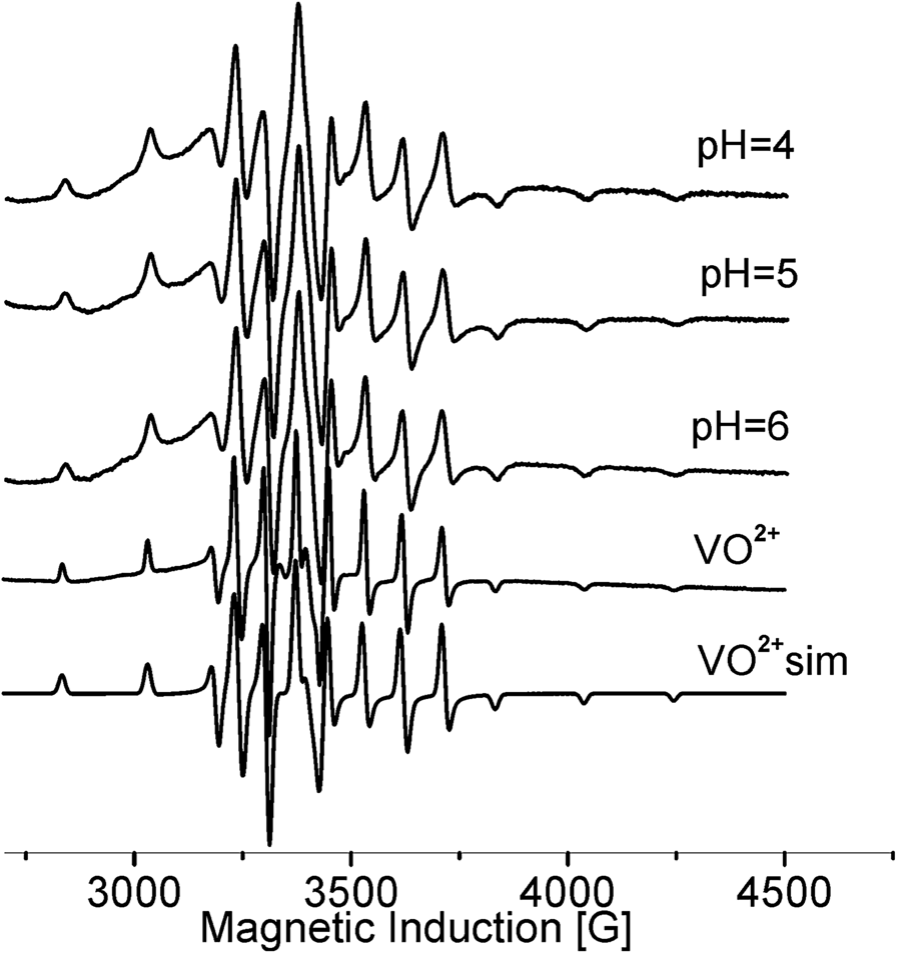
**EPR spectra of aqueous frozen solutions containing VOSO**_**4**_ **and chitosan as a function of pH together with the spectrum obtained (VO** ^**2**^ **sim) by computer simulation of the experimental spectrum of water solution of VOSO**_**4**_ **.**

The EPR spectra of water frozen solutions of VOSO_4_ – chitosan system (Figure 6), exhibit eight line hyperfine splitting due to I (^51^ V) I = 7/2. The parallel hyperfine lines are located symmetrically around *g*|| = 1.933 (with *A*|| ~ 180 ×10^−4^ cm^−1^) whereas the perpendicular lines around *g*_⊥_ = 1.977 (with *A*_⊥_ = ~ 70 ×10^−4^ cm^−1^). The EPR parameters, *g*|| < *g*_⊥_, correspond to axial symmetry of compressed tetragonal geometry of VO^2+^ complexes. On the other hand the relation *g*|| < *g*_⊥_ < 2.0023 is an unambiguous proof of *d*^1^ electron configuration of vanadium ion. The EPR *g* and ***A*** tensor components corresponding to *H* = *β****S g B*** and *H* = ***S A I*** spin Hamiltonians of the VO(IV) complexes formed at various pH are almost similar to those observed for water solution of VOSO_4_. This indicates that amine nitrogen atoms of chitosan are not involved in VO(IV) coordination and supports that only oxygen donors of hydroxyl groups participate in VO(IV) complexation, as it has been already shown by the potentiometric results.

## Conclusions

The studies show that MCCh interacts with oxovanadium(IV) in aqueous solution starting from pH 5. Then, the deprotonation of amine groups is followed by deprotonation of the hydroxyl groups, which leads to a higher use of alkali in the pH-potentiometric titration with respect to chitosan alone, proportional to the part of vanadyl ion in the sample. Thus, the refinements based on potentiometric data confirm coordination via oxygen atoms from the deprotonated hydroxyl groups. From the coordination modes accepted: OH deprotonated MLH_−1_^−^ and twice OH deprotonated ML_2_H_−2_^2−^ species, the second one involves a moiety with one vanadyl binding two adjacent chitosan mers, most probably of two neighbouring polymer chains. Lack of complexation via deprotonation of amine groups, typical for simple cations and the molybdenum anion, has been indicated also by FTIR spectroscopy and EPR. It seems then quite understandable that the degree of deacetylation has no essential influence on the thermodynamical stability of the MLH_−1_^−^ species predominating within the main range of complexation. On the other hand the second deprotonated species i.e. ML_2_H_−2_^2−^ is formed when the titrations are evidently disturbed by increasing aggregation of MCCh regardless DD.

## Methods

### Materials

MCCh (weight-average molecular weight *M*_w_ = 2⋅10^5^ Da, Institute of Biopolymers and Chemical Fibers, Łódź, Poland) was used in the form of hydrogel of definite polymer content (2.55 and 2.56 wt%) at two different average degrees of DD: 79.8, 97.7%. The degree of DD, necessary to estimate the content of –NH_2_ groups in the samples, was determined by the method of first derivative UV-spectrophotometry (1DUVS) according to Khor and co-workers [33]. Vanadium(IV) stock solution was prepared from VOSO_4_ × H_2_O (Alfa Aesar GmbH & Co KG). Carbonate-free 0.1000 ± 0.0003 M NaOH solution (Mallinckrodt Baker B.V.) was used as titrant. Other reagents, i.e., nitric acid and potassium nitrate were from grade pro analysis (P.A.).

### Potentiometric titrations

A Molspin instrument (Newcastle upon Tyne, England) equipped with an OSH-10-10 combined electrode (METRON, Poland), and autoburette was used for EMF measurements. All titrations were run at least in duplicate to ensure reliability of the data. The total volume of the Hamilton microsyringe in the autoburette amounted to 500 μL. The number of up to 100 titration points was attained by volume increments 5.0 μL. The titration course was controlled by MOLSPIN software. All the measurements were carried out at 25.0 ± 0.1°C and ionic strength 0.1 mol L^−1^ (KNO_3_). The cell was standardized according to IUPAC recommendations with two buffers: pH = 3.926 (potassium hydrogen phthalate 0.05 M + 0.05 M KNO_3_) and pH = 9.10 (disodium tetraborate 0.01 M + 0.07 M KNO_3_) [34]. It follows then, that the buffer solutions were of the same ionic strength, medium and temperature as in the tested solutions. In addition, prior to each titration the electrode system had been calibrated in the –log [H^+^] scale by strong acid-strong base titrations, according to the procedure recommended by Irving et al. [35]. In particular, 0.005 M HNO_3_ (adjusted to *I* = 0.1 mol L^−1^ by adding KNO_3_) was neutralized with carbonate-free 0.1 M NaOH at temperature 25.0 ± 0.1°C. The ionization product of water (*pK*_*w*_) under these conditions was 13.77 in our study and was in accordance with the references [36]. The values of standard electromotive force (*E*_0_), which also comprises the liquid junction potential, and slope (*s*) from the equation *E = E*_0_*- s*⋅59.16(−log [H^+^]) were evaluated by Superquad and Hyperquad 2008 [37-39]. The parameters, that were different from the ones obtained from the two-point cell standardization on pH, were then inserted into the input files of the programs Superquad and Hyperquad 2008 to evaluate the overall, concentration formation constants: *β*_*mlh*_ = [M_*m*_L_*l*_H_*h*_]/[M]^*m*^[L]^*l*^[H]^*h*^, where: M = VO^2+^, L = microcrystalline chitosan (MCCh), H = hydrogen (proton). Goodness-of-fit was tested by two parameters: *σ* (connected with the objective function $U={\displaystyle \sum_{i=1}^n{W}_i{r_i}^2},$ where *W*_*i*_ – weight of the *i*-th experimental point of *n* and *r*_*i*_ – *i*-th residual in EMF (*E*_*exp*_ - *E*_*theoret*_)) as well as by the χ^2^ statistics (test of randomness).

### FTIR spectrophotometric measurements

Polymer films in absence and presence of the vanadyl ion were prepared for the use in IR studies. In the latter case the optimum L:M ratio was 5:1 just as in the potentiometric measurements. Initially a portion of 0.075 mmole of nitric (V) acid was added to 0.070 mmole of MCCh to dissolve the ligand. By using 0.1 M NaOH each sample was brought to definite pH within the range 3.0 - 6.8. The formed water slurry was put on a teflon plate and left drying at room temperature. Then the polymer film was removed and used in the FTIR measurements on a Perkin Elmer FT-IR System Spectrum BX spectrophotometer. A total of 10 scans were accumulated. Spectral resolution was ±4 cm^−1^. For comparison polymer membranes with chitosan in absence of the metal were prepared as well. Separately, the IR spectra of VO^2+^ ion were taken in KBr pellets.

### EPR measurements

The EPR spectra were measured using a Bruker Elexsys E500 spectrometer equipped with NMR teslameter (ER 036TM) and frequency counter (E 41 FC) at X-band. The simulations of the experimental spectra were performed using computer program WINEPR Simfonia, version 1.26 beta and the program written by Dr Andrew Ozarowski from NHMFL, University of Florida, with resonance field calculated by diagonalization of energy matrix. The spectra were measured with a modulation frequency 100 kHz, modulation amplitude of 7 gauss and microwave power of 10 mW. The MCCh ligand (L) and VO^2+^ concentrations were *C*_L_ = 7.0 × 10^−3^ M and *C*_VO2+_ = 8.75 × 10^−4^ M, respectively. In order to avoid aggregation of VO(IV) complexes in water 10% (v/v) of ethyl glycol was added to the studied solutions.

## Abbreviations

DD: Degree of deacetylation
FTIR: Fourier-transform infrared spectroscopy
EPR: Electron paramagnetic resonance
L = MCCh: Microcrystalline chitosan as ligand
M: Oxovanadium (IV) ion as metal
L/M: The ligand-to-metal concentration ratio
*a*: mmole of base/mmole of ligand
*β*_*mlh*_ = [M_*m*_L_*l*_H_*h*_]/[M]^*m*^[L]^*l*^[H]^*h*^: Cumulative stability constant
*m, l*, *h*: Number of metals (central ions), ligands and protons, respectively; concentrations in square brackets are equilibrium concentrations
